# Interrelation between homocysteine metabolism and the development of autism spectrum disorder in children

**DOI:** 10.3389/fnmol.2022.947513

**Published:** 2022-08-15

**Authors:** Bingbing Li, Yiran Xu, Dizhou Pang, Qiang Zhao, Lingling Zhang, Ming Li, Wenhua Li, Guiqin Duan, Changlian Zhu

**Affiliations:** ^1^Henan Key Laboratory of Child Brain Injury and Henan Pediatric Clinical Research Center, Third Affiliated Hospital and Institute of Neuroscience, Zhengzhou University, Zhengzhou, China; ^2^Center for Child Behavioral Development, Third Affiliated Hospital of Zhengzhou University, Zhengzhou, China; ^3^Key Clinical Laboratory of Henan Province, Department of Clinical Laboratory, First Affiliated Hospital of Zhengzhou University, Zhengzhou, China; ^4^Center for Brain Repair and Rehabilitation, Institute of Neuroscience and Physiology, University of Gothenburg, Sahlgrenska Academy, Gothenburg, Sweden

**Keywords:** homocysteine, folate, vitamin B12, autism spectrum disorder, biomarkers

## Abstract

Evidence is emerging that dysregulation of circulating concentrations of homocysteine, an important intermediate in folate and vitamin B12 metabolism, is associated with autism spectrum disorder (ASD), but comprehensive assessments and correlations with disease characteristics have not been reported. Multivariate ordinal regression and restricted cubic spline (RCS) models were used to estimate independent correlations between serum homocysteine, folate, and vitamin B12 levels and clinical outcomes and severity of children with ASD. After adjusting for confounding factors, serum homocysteine levels were significantly higher in children with ASD than in healthy controls (β: 0.370; 95% CI: 0.299~0.441, *p* < 0.001). Moreover, homocysteine had a good diagnostic ability for distinguishing children with ASD from healthy subjects (AUC: 0.899, *p* < 0.001). The RCS model indicated a positive and linear association between serum homocysteine and the risk of ASD. The lowest quartile of folate was positively associated with ASD severity (OR: 4.227, 95% CI: 1.022~17.488, *p* = 0.041) compared to the highest quartile, and serum folate showed a negative and linear association with ASD severity. In addition, decreased concentrations of folate and vitamin B12 were associated with poor adaptive behavior developmental quotients of the Gesell Developmental Schedules (*p* < 0.05). Overall, an increased homocysteine level was associated with ASD in a linear manner and is thus a novel diagnostic biomarker for ASD. Decreased concentrations of folate and vitamin B12 were associated with poor clinical profiles of children with ASD. These findings suggest that homocysteine-lowering interventions or folate and vitamin B12 supplementation might be a viable treatment strategy for ASD.

## Introduction

Autism spectrum disorder (ASD) is an early-onset neurodevelopmental disorder characterized by problems with social communication, repetitive behavior, and restricted interests, and patients can manifest a comprehensive array of additional symptoms such as intellectual disabilities and gastrointestinal disorders (Lai et al., 2014). Although heredity is widely recognized as one of the major causes of ASD (Sandin et al., 2017), it cannot explain the recent increase in ASD prevalence (Maenner et al., 2020). Therefore, more and more attention has been paid to the study of environmental factors in the etiology of ASD.

Homocysteine, a sulfur-containing amino acid, is a metabolic intermediate in methionine metabolism and is crucial for methionine availability, nucleotide synthesis, and methylation reactions (Koklesova et al., 2021). Elevated serum homocysteine levels have been identified as a risk factor for several neurological and psychiatric disorders, including cognitive impairment (Zhou et al., 2021), dementia (Chen et al., 2020), and bipolar disorders (Salagre et al., 2017), and they have been shown to have direct neurotoxicity and to induce oxidative stress and mitochondrial dysfunction, both of which are implicated in ASD (Frye et al., 2013a; Balachandar et al., 2021). These observations have raised questions regarding whether elevated serum homocysteine is a biomarker or a therapeutic target of ASD. However, previous observational studies generated contrasting results, with some showing significant positive associations (Altun et al., 2018; Chen et al., 2021) and others showing no associations (Main et al., 2015; Saha et al., 2022). More importantly, no studies assessed the specific shape of this association while controlling for confounding factors that might affect the serum concentrations of homocysteine such as age, sex, and the use of vitamin supplements. All these factors might lead to some bias and preclude an exhaustive evaluation of the role of homocysteine in ASD. Also, prior studies did not fully examine the relationship between homocysteine levels and clinical severity of ASD symptoms and neurodevelopment in children with ASD.

In addition, levels of homocysteine are inversely correlated with levels of folate and vitamin B12 in the blood (Refsum et al., 2004), which are cofactors for the folate-dependent methylation of homocysteine to methionine (Škovierová et al., 2016) and are required for the synthesis of both phospholipids and myelin, and are crucial for the normal functioning of the brain (Hassan et al., 2019). Folate and vitamin B12 deficiencies have been linked to cognitive impairment (Dror and Allen, 2008; Michelakos et al., 2013) and ASD (Al-Farsi et al., 2013; Li et al., 2021). Moreover, 38% of children with ASD have cerebral folate deficiency syndrome (Rossignol and Frye, 2021a), which is a new emerging group of syndromes that are characterized by low cerebral folate levels under normal blood folate levels. The most common cause of low cerebral folate in this syndrome is the presence of folate receptor autoantibodies that can block folate transport across the blood–brain barrier to the brain and thus influence the development of the central nervous system (Ramaekers et al., 2005; Bobrowski-Khoury et al., 2021). Animal models demonstrated that a decrease in brain folate resulted in homocysteine accumulation and changes in neuronal excitability and maintenance, which contributed to the development of cognitive deficits (Kruman et al., 2005; Mann et al., 2018). Furthermore, owing to gastrointestinal disorders and/or picky eating (Sharp et al., 2013), children with ASD often have inadequate dietary intakes, leading to severe vitamin B12 and folate deficiencies (Ranjan and Nasser, 2015), which might further increase serum homocysteine levels and worsen autism symptoms. It can thus be debated whether it is low levels of folate or vitamin B12 that play an important role in the etiology of ASD. Thus, more extensive well-designed studies are needed to clarify this association.

The primary aim of the present study was to comprehensively investigate the relationship of homocysteine levels with the risk and clinical severity of ASD and its potential role as a diagnostic marker of the risk of developing ASD or for predicting the severity of ASD, which may pave the way for new therapeutic strategies for ASD. Owing to the interrelatedness of homocysteine, folate, and vitamin B12 levels as discussed earlier, we additionally examined the associations between serum folate and vitamin B12 levels and the risk and severity of ASD.

## Methods

### Participants

In this case–control study, 135 patients who were diagnosed with ASD were consecutively recruited from the inpatient department of the Third Affiliated Hospital of Zhengzhou University from August 2019 to November 2021. The inclusion criteria were age from 1 year to 6 years, patients having completed questionnaires on diet and medical history, those having anthropometric measurements recorded, and those not having consumed vitamin B for more than 3 months prior to the blood sample collection. Children with other developmental disorders or psychiatric diseases such as Rett syndrome, cerebral palsy, or chronic seizures were excluded from the study. A total of 84 healthy individuals were recruited from the community as volunteers with their parents' consent, and these individuals had no physical or neurological disorders and had not received vitamin B supplementation in the previous 3 months. This study was designed and conducted in accordance with the Declaration of Helsinki. All subjects were recruited during the same period, and written informed consent was obtained from the primary caregivers of all participants. The study protocol was approved by the ethics committee of the Third Affiliated Hospital of Zhengzhou University (Approval # 2020-126-01).

### Data collection

Information on demographics, dietary preference for meat and vegetables, medical history, and family or personal history from the two groups of children was collected by well-trained nurses using a pre-tested questionnaire. Anthropometric assessments were performed by trained health professionals following a standardized protocol. The body mass index (BMI) was calculated and expressed as kg/m^2^. The z-score of BMI-for-age (ZBMIA) was calculated, and overweight was defined as a ZBMIA > 2 according to the WHO criteria (WHO, 2008). Information on the history of vitamin supplements was recorded because this could affect the participants' levels of homocysteine, folate, and vitamin B12.

### Diagnosis and behavioral assessments

Children in the ASD group were diagnosed by using the Diagnostic and Statistical Manual of Mental Disorders, 5th Edition (Regier et al., 2013) for the first time without systemic intervention, and ASD symptoms were assessed by using the Childhood Autism Rating Scale (CARS) by two experienced psychiatrists (Schopler et al., 1980). CARS is an assessment scale of the severity of autistic symptomatology and consists of 15 items that are rated on a four-point scale, and the higher the score, the more severe the symptoms. Children with scores between 30 and 36 are considered to having mild to moderate autism, and those with scores between 37 and 60 are considered severe autism. Neurodevelopmental levels of children with ASD were evaluated using the Gesell Developmental Schedules (GDS), which have been widely used to evaluate the development of children aged from 16 days to 6 years (Liu et al., 2016, 2022). The GDS include five subscales, namely, adaptive, gross motor, fine motor, language, and personal–social behavior. The developmental quotient (DQ) of the five domains was used to evaluate the level of neurodevelopment, and a DQ ≤ 75 for two or more subscales of the GDS indicates a developmental delay.

### Sample collection and measurements

A measure of 2 mL of venous blood was drawn into a serum separation tube and immediately centrifuged (1,500 × g for 5 min) to separate the serum from the cells, after which 0.2 mL of serum was pipetted into several vials and stored at −80°C until being assayed. Serum homocysteine levels were measured using the enzyme cycling assay (Roche Diagnostics, Cobas e 801, Switzerland), and serum folate and vitamin B12 levels were analyzed using automated electrochemiluminescence immunoassays (Roche Diagnostics, Cobas 8000, Switzerland).

### Statistics

Statistical analyses were performed using SPSS version 23 (SPSS Inc., Chicago, IL, USA), SAS version 14 (SAS Institute, Cary, North Carolina, USA), and Stata version 12 (Stata Corporation, College Station, TX, USA). The characteristics of all the subjects are presented as numbers (percentages) for categorical variables, as means ± standard deviations for parametrically distributed variables or as medians (interquartile ranges) for non-parametrically distributed variables. The differences between the ASD group and control group were analyzed by using the Student's *t*-test or the Wilcoxon rank sum test for comparisons of the continuous variables according to the data distribution and by using the chi-squared tests for the categorical variables. The Spearman correlation test was applied to examine the correlations between serum homocysteine, folate, and vitamin B12 levels and the neurodevelopmental level of children with ASD.

Univariate and multivariate linear regression models were applied to compare serum homocysteine, folate, and vitamin B12 levels between the ASD group and the control group. The confounding factors adjusted in the multivariate linear regression models were as follows: model 1 included age, sex, BMI, picky eating, and use of vitamins, and model 2 included all the covariates in model 1 and was additionally adjusted for the combined effects of serum homocysteine, folate, or vitamin B12 levels, which were included in the linear model as continuous variables and were natural logarithm-transformed in order to meet normal distribution.

Multivariable regression analysis models were used to assess the correlations between serum homocysteine and folate levels and the severity of ASD symptoms in children with ASD after adjusting for age, sex, BMI, picky eating, use of vitamins, and serum homocysteine, folate, or vitamin B12 levels (continuous, ln-transformed). The trends across the quartiles of serum homocysteine and folate levels were tested by treating the quartiles as a continuous variable and assigning the midpoint concentration for each quartile. The RCS model was used to show the shape of these associations between homocysteine levels and ASD and between homocysteine and folate levels and the severity of ASD symptoms, respectively. Spearman's correlation analysis and multivariate linear regression models were used to assess the relationship between serum homocysteine, folate, and vitamin B12 levels and neurodevelopmental outcomes in children with ASD.

Cumulative frequency distributions were calculated to show the distribution of serum homocysteine levels in children with ASD and healthy controls. Finally, the predictive value of serum homocysteine, folate, and vitamin B12 levels and their combination for developing ASD was analyzed by receiver operating characteristic (ROC) analysis. A two-tailed p-value < 0.05 and a 95% CI not covering the null value were considered statistically significant for all analyses.

## Results

### Characteristics of the participants

**Figure 1:**
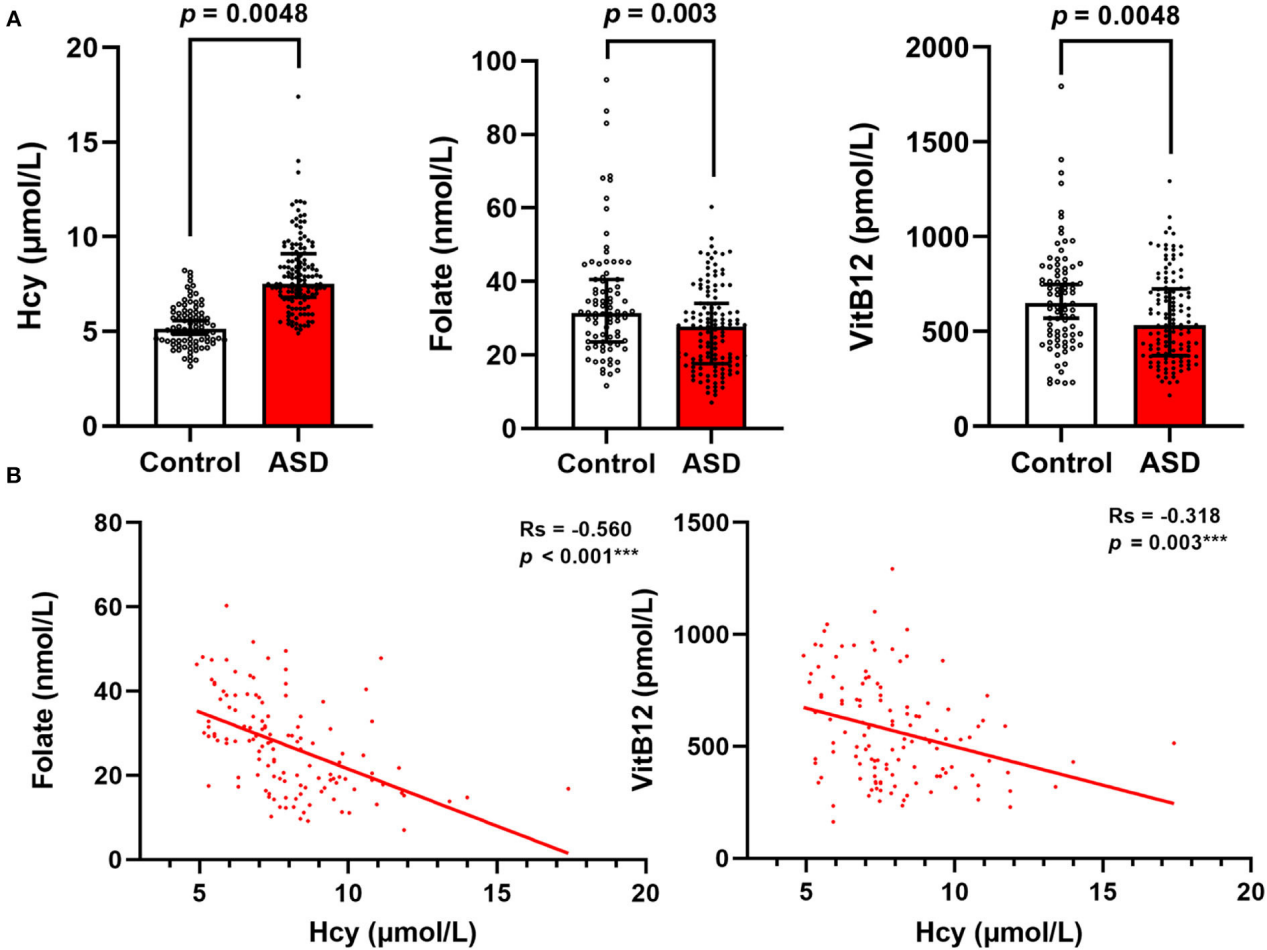
Serum Hcy, folate, and VitB12 levels differ between children with ASD and healthy controls. **(A)** Comparison of Hcy, folate, and VitB12 levels between the two groups. **(B)** Correlations between serum Hcy, folate, and VitB12 levels in children with ASD. Results are presented as Spearman correlation coefficients (*r*_s_) and p-values. Hcy, homocysteine; VitB12, vitamin B12.

The general characteristics and serum homocysteine, folate, and vitamin B12 concentrations of the study population are shown in Table 1. There were no significant differences in age or sex between the children with ASD and healthy controls (*p* > 0.05). The children with ASD had significantly higher values of BMI (16.39 ± 1.90 vs. 15.59 ± 1.81) and a higher prevalence of overweight (9.6 vs. 3.6%), food selectivity (79.4 vs. 20.6%), resistance to vegetables (81.4 vs. 18.6%), and history of taking vitamins (82.6 vs. 17.4%) than the healthy controls. Moreover, 51.9% of the children with ASD had severe symptoms of ASD, and 85.2% had developmental delays. The serum levels of homocysteine were significantly higher (*p* = 0.0048) in the ASD group than in the control group, while the serum levels of folate (*p* = 0.003) and vitamin B12 (*p* = 0.0048) were lower in the ASD group (Figure 1A). The Spearman correlation coefficient (*r*_s_) between serum homocysteine and folate was −0.560 and that for vitamin B12 was −0.318 (Figure 1B).

**Table 1:** General characteristics of the participants in the study.

**Variables**	**Total population** ** (*n* = 219)**	**ASD** ** (*n* = 135)**	**Controls** ** (*n* = 84)**	***p*-value**
**Age, years**	3.25 (3.00,3.83)	3.25 (2.75,4.25)	3.33 (3.50,3.00)	0.647
**≤3**	82 (37.4)	55 (40.7)	27 (32.1)	0.251
**>3**	137 (62.6)	80 (59.3)	57 (67.9)	–
**Men, n (%)**	181 (82.60)	117 (86.70)	64 (76.20)	0.066
**BMI, kg/m** ^**2**^	16.09 ± 1.90	16.39 ± 1.90	15.59 ± 1.81	<0.001
**ZBMIA**	0.38 ± 1.40	0.65 ± 1.33	−0.05 ± 1.40	0.001
**Overweight**	26 (11.87)	23 (9.6)	3 (3.6)	0.002
**Developmental delay**, ***n*** **(%)**	115 (52.51)	115 (85.2)	0	–
**Picky eating**, ***n*** **(%)**	97 (44.29)	77 (79.40)	20 (20.60)	0.001
**Resistance to vegetables**, ***n*** **(%)**	70 (31.96)	57 (81.4)	13 (18.6)	<0.001
**Resistance to meats,** ***n*** **(%)**	36 (16.44)	27 (75.0)	9 (25.0)	0.091
**Resistance to fruits,** ***n*** **(%)**	14 (6.40)	11 (78.60)	3 (21.40)	0.258
**History of taking vitamins**, ***n*** **(%)**	86 (39.27)	71 (82.6)	15 (17.4)	<0.001
**Severity of ASD**, ***n*** **(%)**	
**Mild to moderate**	65 (29.68)	65 (48.1)	0	–
**Severe**	70 (31.96)	70 (51.9)	0	–
**Hcy**, **μmol/L**	6.68 (5.30, 7.95)	7.50 (6.70, 8.90)	5.15 (4.49, 6.03)	0.005
**Folate, nmol/L**	29.3 (19.6, 37.7)	27.6 (17.6, 34.8)	31.4 (23.5, 40.5)	0.003
**VitB12, pmol/L**	583 (415, 772)	530 (369, 708)	649 (478, 839)	0.005

*BMI, body mass index; ZBMIA, z score for BMI-for-age; ASD, autism spectrum disorder; Hcy, homocysteine; VitB12, vitamin B12. Overweight is defined as a ZBMIA > 2. Data are presented as number (percentage) for categorical data, mean ± standard deviation for parametrically distributed data, or median (interquartile range) for non-parametrically distributed data. Student's t-test or the Wilcoxon rank sum test was used for comparison of the continuous variables according to the data distribution, and chi-square tests were used for the categorical variables*.

### Association of serum homocysteine, folate, and vitamin B12 levels in children with ASD

**Figure 2:**
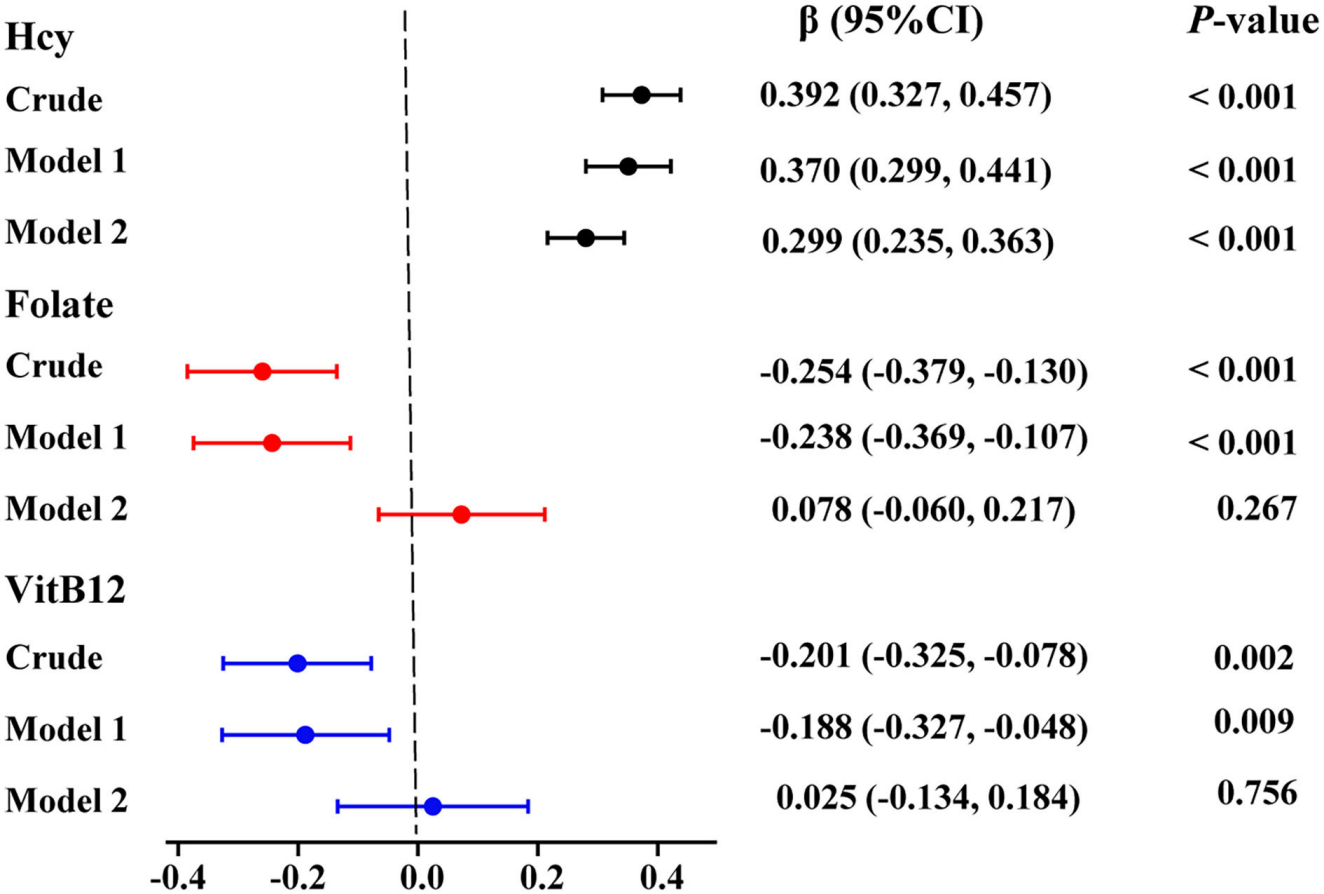
Multivariate linear logistic regression analysis for the associations between serum Hcy, folate, and VitB12 levels and ASD. Colored points represent the adjusted regression coefficient (β), and horizontal lines represent the 95% CI. “Crude” means no adjustment. Model 1 was adjusted for age, sex, BMI, picky eating, and use of vitamins. Model 2 was adjusted for model 1 and the other two metabolites. Hcy, homocysteine; VitB12, vitamin B12.

We used linear regression models to analyze the correlation of serum homocysteine, folate, and vitamin B12 with ASD. As seen in Figure 2 and Supplementary Table 1, after adjusting for age, sex, BMI, picky eating, and history of use of vitamins in model 1, high levels of serum homocysteine (β: 0.370; 95% CI: 0.299 ~ 0.441, *p* < 0.001) and low levels of serum folate (β: −0.238; 95% CI: −0.369 ~ −0.107, *p* < 0.001) and vitamin B12 (β: −0.188; 95% CI: −0.327 ~ −0.048, *p* = 0.009) were significantly associated with ASD. Notably, increased levels of serum homocysteine (β: 0.299; 95% CI: 0.235 ~ 0.363, *p* < 0.001) remained significantly correlated with ASD even after successively adjusting for serum folate and vitamin B12 levels in model 2. However, the associations between low serum folate levels and vitamin B12 levels were no longer significant after adjusting for all confounding variables in model 2.

**Figure 3:**
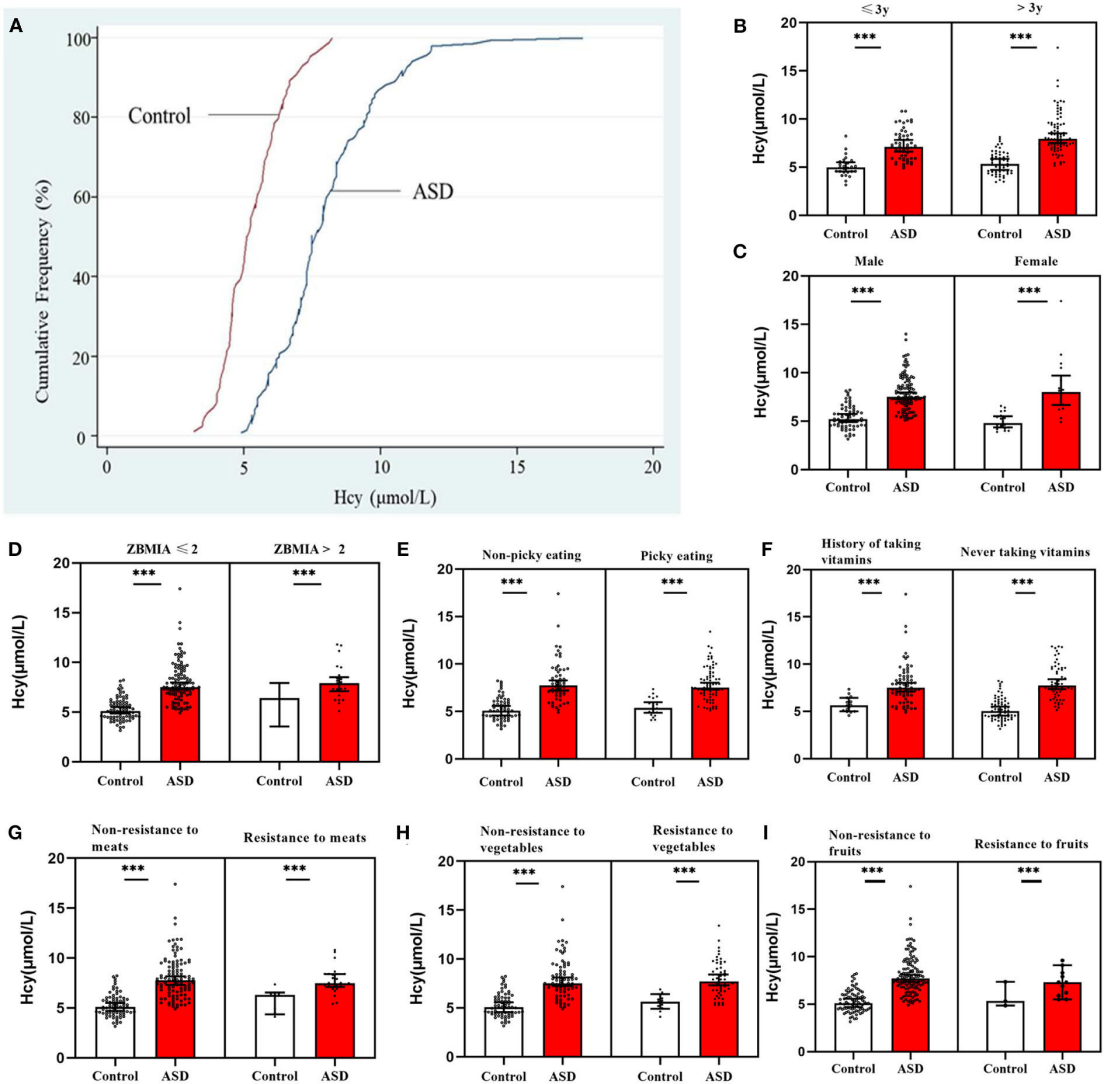
Serum homocysteine (Hcy) levels in children with ASD and controls. **(A)** Cumulative frequency distributions of Hcy levels in both groups. **(B–I)** Serum Hcy levels were stratified by age, sex, BMI, food selectivity, and use of vitamins. In both groups, there were significant differences between serum Hcy levels in children with ASD for each covariate (***p < 0.001). Hcy, homocysteine; VitB12, vitamin B12.

The cumulative frequency distribution of serum homocysteine concentrations in the ASD and control groups is shown in Figure 3A. There was a clear distinction between the two groups, and there was a marked increase in the distribution of the homocysteine concentration in children with ASD compared with healthy controls. Furthermore, in the subgroup analyses, sex, age ( ≤ 3 years vs. > 3 years), ZBMIA ( ≤ 2 vs. > 2), dietary preference for meat and vegetables, and use of vitamins in the children with ASD all exhibited significantly higher serum homocysteine levels than healthy controls for each covariate (all *p* < 0.05, Figures 3B–I).

### The ROC analysis of serum homocysteine, folate, and vitamin B12 to distinguish children with ASD from healthy controls

**Figure 4:**
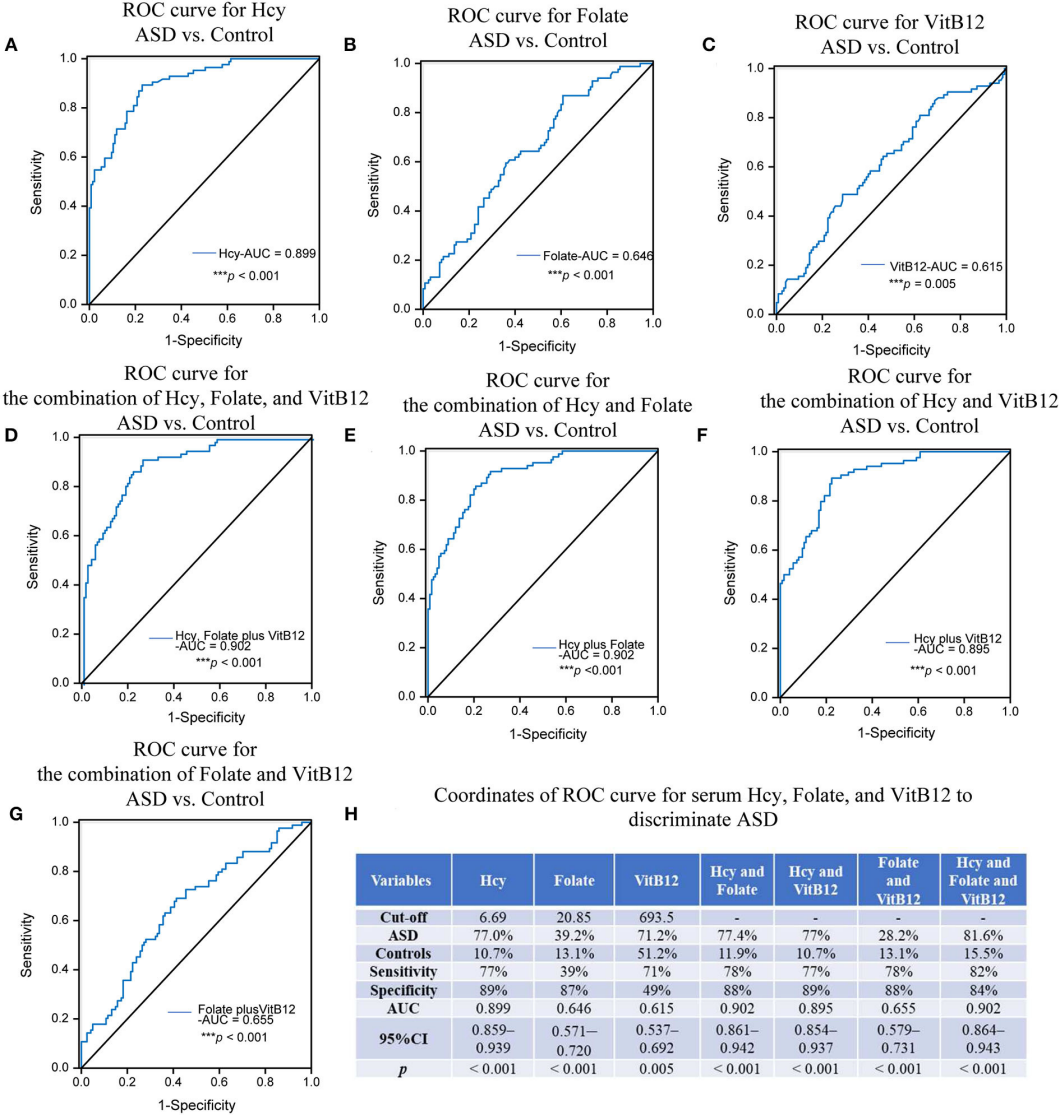
ROC analysis of Hcy, folate, and VitB12. The AUCs of the ROC curves for **(A)** Hcy, **(B)** folate, and **(C)** VitB12 were 0.899 (***p < 0.001), 0.646 (*p* = 0.230), and 0.615 (***p < 0.001), respectively. The AUCs of the ROC curves for **(D)** Hcy+folate+VitB12, **(E)** Hcy+folate, **(F)** Hcy+VitB12, and **(G)** folate+VitB12 were 0.902 (***p < 0.001), 0.902 (***p < 0.001), 0.895 (***p < 0.001) and 0.655 (***p < 0.001), respectively. **(H)** Coordinates of ROC curve for Hcy, folate, and VitB12 to discriminate ASD from heathy controls. Hcy, homocysteine; VitB12, vitaminB12.

The optimum cutoff points for serum homocysteine, folate, and vitamin B12 levels were set at the maximum value of the Youden index. These values are presented in Figure 4, along with the area under the ROC curve (AUC), sensitivity and specificity in detecting ASD, and the associated p-values. The AUC of homocysteine was 0.899 (95% CI: 0.859 ~ 0.939), and the cutoff was 6.69 μmol/L (sensitivity: 77%, specificity: 89%), which indicated a relatively high discrimination ability. Because the AUCs of folate and vitamin B12 were 0.646 and 0.615, respectively, they could not differentiate the children with ASD from the healthy controls. Furthermore, the AUCs of the combination of homocysteine and folate with or without vitamin B12 were both 0.902, which was slighter higher than the AUC of serum homocysteine alone, thus showing better diagnostic ability. This indicates that the diagnostic performance of serum homocysteine alone was equivalent to that of the combination of the three biomarkers, suggesting that homocysteine alone might be a potential biomarker for ASD.

### Association of serum homocysteine, folate, and vitamin B12 levels with ASD severity

The baseline demographic, clinical, and laboratory characteristics of the children with ASD grouped by the severity of ASD symptoms according to CARS scores are given in Table 2. The median values of serum homocysteine levels increased significantly across the groups of ASD severity, while the median values of serum folate levels decreased significantly across the groups of ASD severity. No significant differences in age, sex, BMI, food selectivity, history of birth asphyxia, or other clinical characteristics were observed between the two groups (*p* > 0.05). In addition, we further analyzed the impact of age, sex, BMI, dietary habits, and comorbidity on serum homocysteine and folate levels in children with ASD. As seen in Supplementary Figure 1, serum homocysteine (*r*_s_ = 0.334, *p* < 0.001) and folate levels (*r*_s_ = −0.438, *p* < 0.001) were associated with age, and children with ASD older than 3 years exhibited higher serum homocysteine and lower folate levels than children with ASD younger than 3 years (*p* < 0.05). Interestingly, serum folate levels were lower in children with ASD who resisted vegetables and who did not take vitamins before this analysis (*p* < 0.05, Supplementary Figure 2). No significant correlations were found for serum homocysteine with dietary preferences in children with ASD.

**Table 2:** Baseline characteristics of patients with ASD grouped by severity of clinical symptoms according to CARS.

**Variables**	**Total** ** (*n* = 135)**	**CARS < 36** ** (*n* = 65)**	**CARS ≥36** ** (*n* = 70)**	***p*-value**
Age, years	3.25 (2.75–4.25)	3.25 (2.75–3.96)	3.29 (2.67–4.50)	0.346
Male, *n* (%)	117 (86.7)	56 (86.2)	61 (87.1)	1.0
BMI, kg/m^2^	16.39 (1.90)	16.56 (2.05)	16.24 (1.75)	0.34
Overweight, *n* (%)	23 (17.0)	14 (21.5)	9 (12.9)	0.252
History of birth asphyxia, *n* (%)	19 (14.1)	10 (15.4)	9 (12.9)	0.805
Natural labor, *n* (%)	61 (45.2)	33 (50.8)	28 (40)	0.229
Birth weight, kg	3.30 (3.10–3.60)	3.30 (2.95–3.58)	3.30 (3.10–3.70)	0.141
History of neonatal care, *n* (%)	15 (11.1)	8 (12.3)	7 (10.0)	0.786
Siblings ≥1, *n* (%)	71 (52.6)	30 (46.2)	41 (58.6)	0.170
Breast-fed, *n* (%)	95 (70.4)	45 (69.2)	50 (71.4)	0.851
Severe jaundice, *n* (%)	11 (8.1)	8 (12.3)	3 (4.3)	0.118
Picky eating, n (%)	77 (57.0)	38 (58.5)	39 (55.7)	0.862
Resistance to vegetables, *n* (%)	57 (42.2)	25 (38.5)	32 (45.7)	0.486
Resistance to meats, *n* (%)	27 (20.0)	14 (21.5)	13 (18.6)	0.674
Resistance to fruits, *n* (%)	11 (8.1)	8 (12.3)	3 (4.3)	0.118
History of taking vitamins, *n* (%)	71 (53.4)	34 (54.0)	37 (52.9)	1.0
Developmental delay, *n* (%)	115 (85.2)	54 (83.1)	61 (87.1)	0.629
Language regression, *n* (%)	15 (11.1)	6 (9.2)	9 (12.9)	0.590
Hcy, μmol/L	7.5 (6.70–8.90)	7.1 (6.20–8.32)	8.24 (7.30–9.51)	0.005
Folate, nmol/L	27.6 (17.6–34.8)	30.6 (20.0–39.4)	22.3 (15.6–29.5)	0.001
VitB12, pmol/L	530 (369–70)	587 (408–783)	444 (342–676)	0.140

*BMI, body mass index; Hcy, homocysteine; VitB12, vitamin B12; ASD, autism spectrum disorder; CARS, Childhood Autism Rating Scale*.

**Figure 5:**
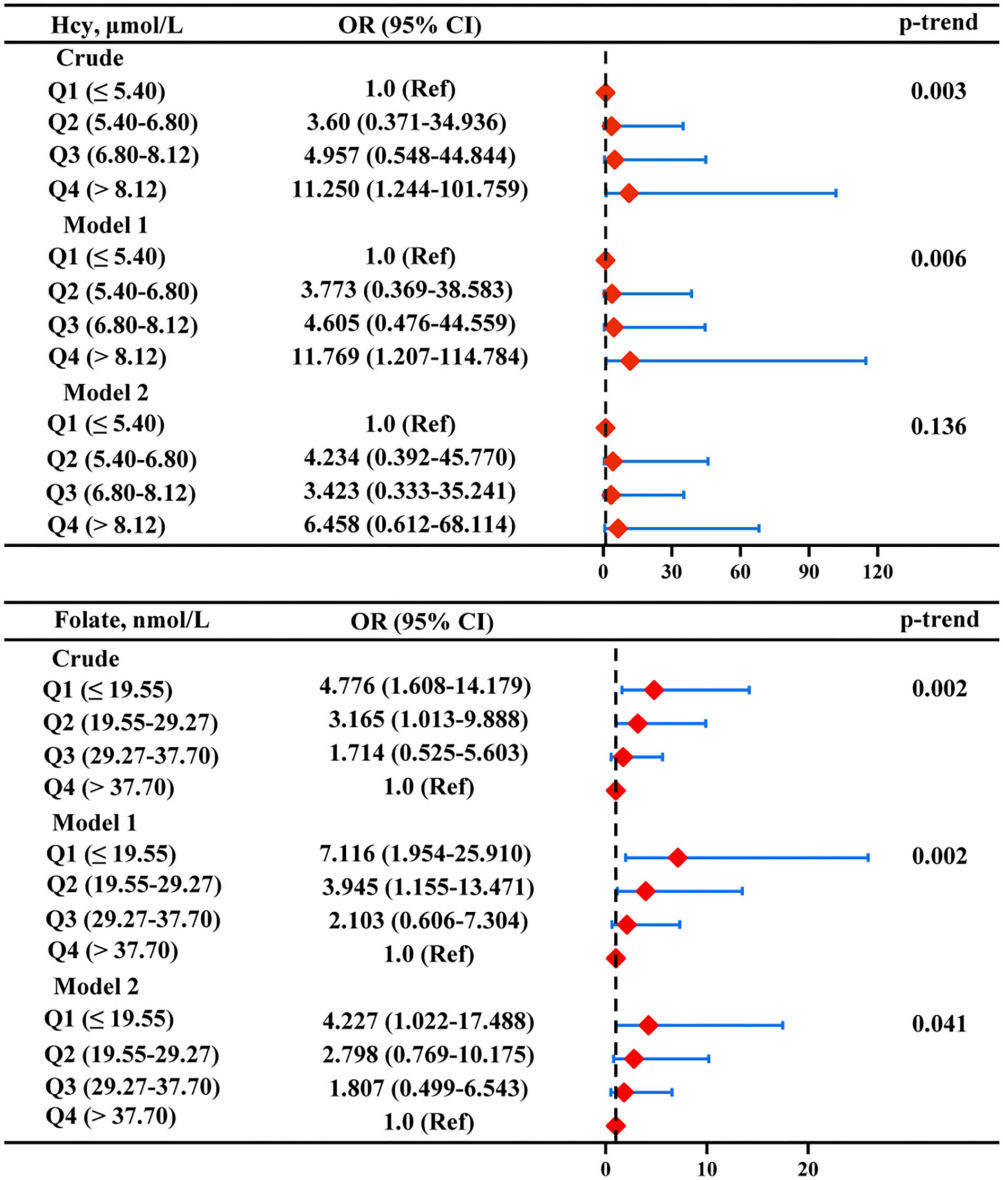
Multiple logistic regression analysis for the associations between serum Hcy and folate and severity of ASD. Model 1 was adjusted for age, sex, BMI, picky eating, and use of vitamins. Model 2 was adjusted for model 1 and the other two metabolites. Red diamonds represent the adjusted OR, and horizon lines represent 95% CI. P-values for the trend test (p-trend) were obtained from the multiple logistic regression models by using the median of each parameter quartile (ln-transformed serum homocysteine and folate concentrations) as a continuous variable. Hcy, homocysteine.

According to the aforementioned results, a further multiple logistic regression analysis was carried out to analyze the correlation between serum homocysteine and folate levels and ASD symptom severity (Figure 5). After adjusting for age, sex, BMI, picky eating, and use of multivitamins (model 1), the highest homocysteine quartile was independently associated with the severity of ASD (OR: 11.769, 95% CI: 1.207 ~ 114.784) compared with the lowest homocysteine quartile. However, the correlation between the highest homocysteine quartile and ASD symptom severity was no longer significant after additionally adjusting for serum folate and vitamin B12 levels in model 2. The multivariate-adjusted OR (95% CI) of ASD severity was 7.116 (1.954 ~ 25.910) for serum folate levels when comparing the lowest versus the highest quartile, and the relationships were still significant after adjustment for all of the confounding factors in model 2 (OR: 4.227, 95%CI: 1.022 ~ 17.488). The linear model also showed a negative association of the serum folate level with ASD severity (*p*-trend < 0.05).

Because we found that folate concentrations were associated with age in children with ASD, an age-stratified analysis was performed to moderate the confounding effect of age. The results showed that serum folate levels were negatively but not strongly associated with the severity of ASD in both age groups. Serum folate levels in the younger age group ( ≤ 3 years) showed a higher tendency for the risk of severe ASD (Supplementary Table 2).

### The RCS analysis of serum homocysteine, folate, and vitamin B12 levels and ASD risk and severity

**Figure 6:**
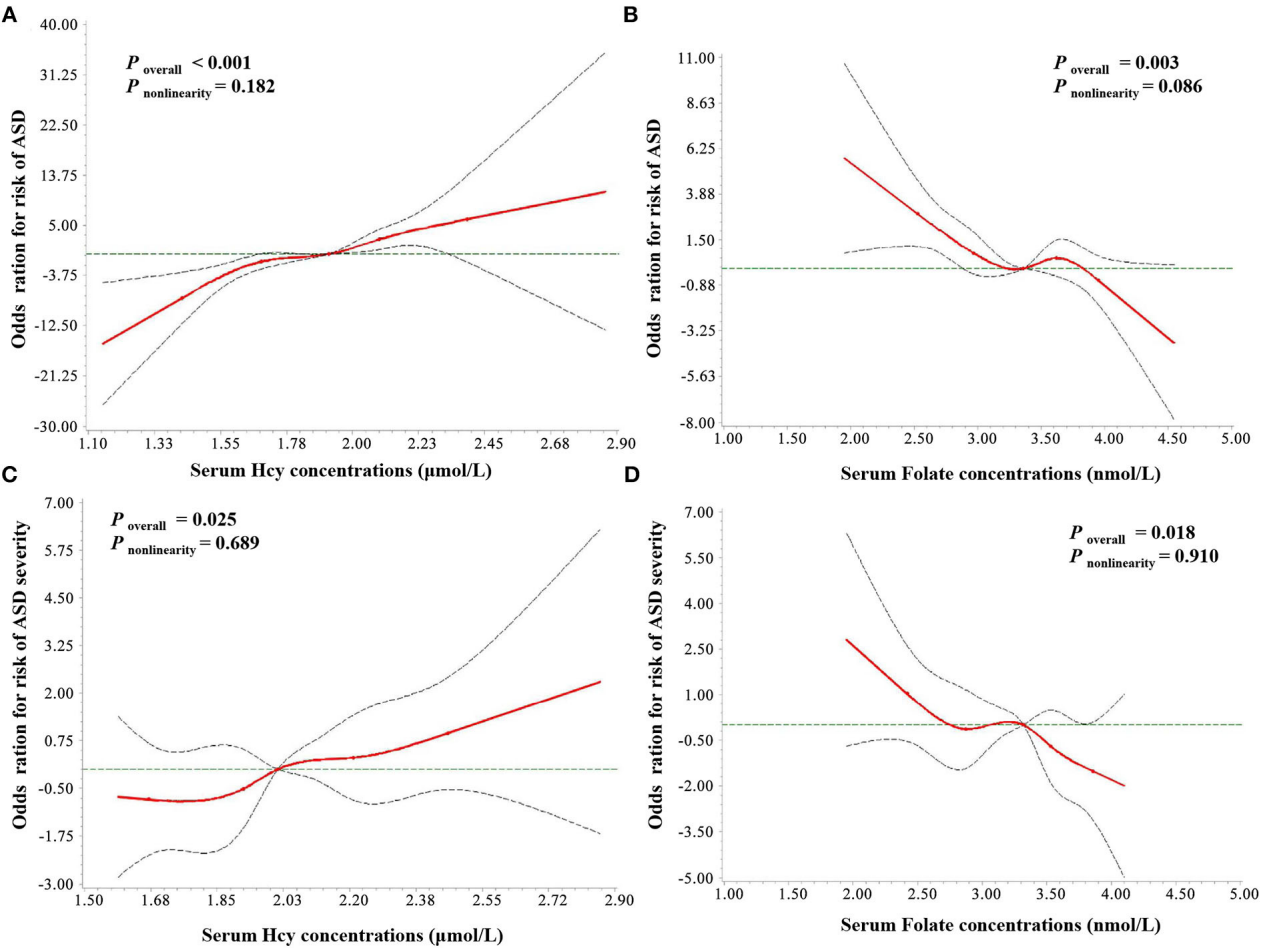
RCS regression analysis of serum Hcy and folate concentrations with ASD risk and severity. The risk estimates for ASD risk **(A,B)** and severity **(C,D)** were adjusted for age, sex, BMI, picky eating, use of vitamins, and the other two metabolites. Lines represent adjusted odds ratios (solid red lines) and 95% CIs (long dashed lines) based on the RCS models for the ln-transformed concentrations of serum Hcy and folate. The reference values were set at the 25th percentiles, and the knots were set at 5th, 25th, 50th, 75th, and 95th percentiles of the ln-transformed concentrations, respectively. Hcy, homocysteine; VitB12, vitamin B12.

As seen in Figure 6, a positive linear association was observed between the serum homocysteine concentration and the risk of ASD (*p*_overall_ < 0.001, *p*_nonlinearity_ = 0.182), while serum folate levels showed a negative linear association with the risk of ASD (*p*_overall_ = 0.003, *p*_nonlinearity_ = 0.086). In addition, the results of RCS regression of ASD severity showed similar linear relationships between serum homocysteine (*p*_overall_ = 0.025, *p*_nonlinearity_ = 0.689) and folate (*p*_overall_ = 0.018, *p*_nonlinearity_ = 0.910) and severity of ASD symptoms. No association was observed between serum vitamin B12 levels and ASD risk (*p*_overall_ > 0.05, *p*_nonlinearity_ = 0.732).

### Associations of serum homocysteine, folate, and vitamin B12 levels with neurodevelopment in children with ASD

**Figure 7:**
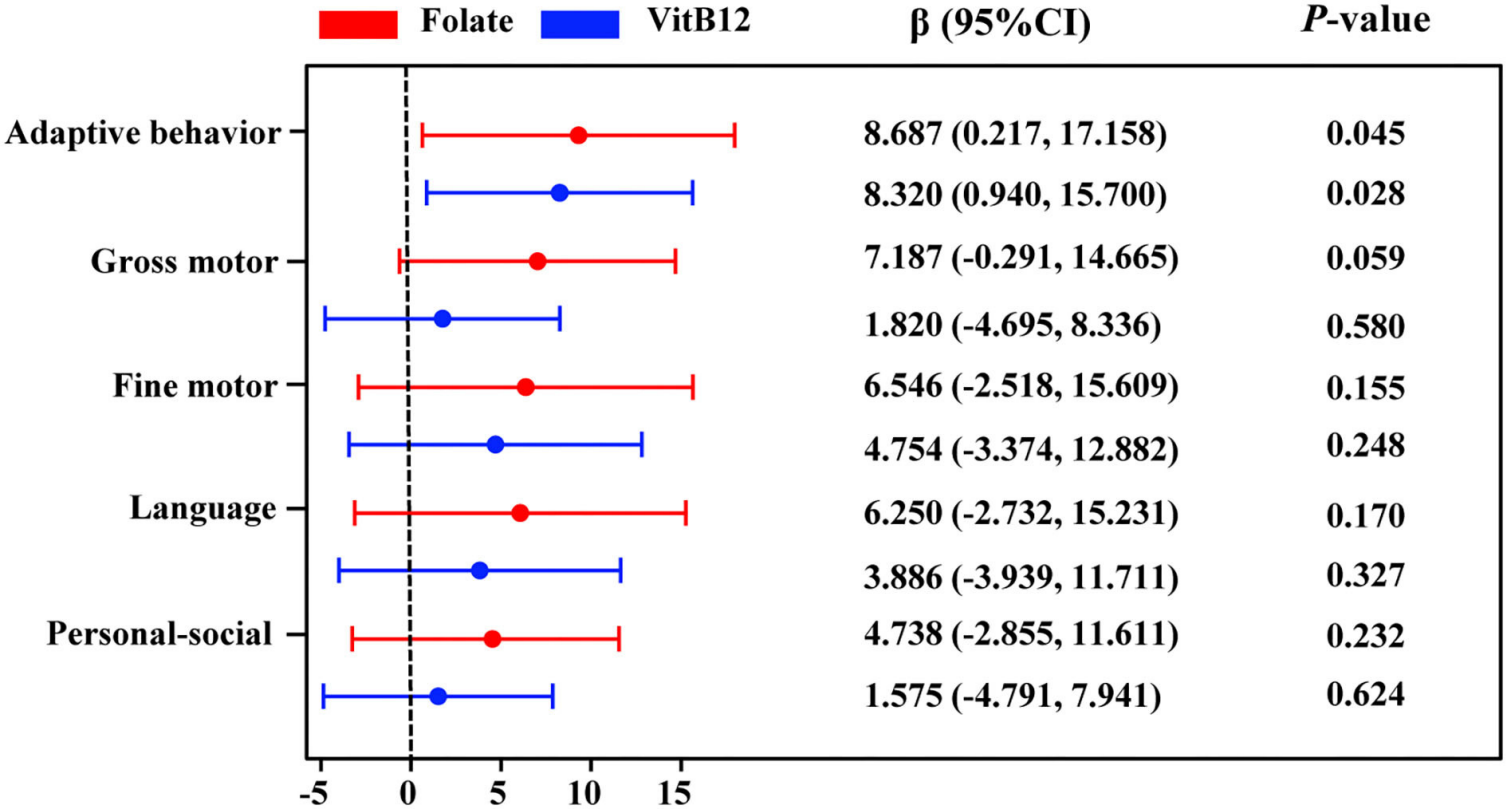
Multiple linear regression for adaptive behavior, gross motor, fine motor, language, and personal–social behavior DQs with serum folate and VitB12 concentrations. Colored points represent the adjusted regression coefficient (β), and horizontal lines represent 95% CI adjusted for age, sex, BMI, picky eating, use of vitamins, and the other two metabolites. VitB12, vitamin B12.

We performed the Spearman correlation analysis to identify the relationship between serum homocysteine, folate, and vitamin B12 levels and Gesell DQ scores in children with ASD. As seen in Supplementary Figure 3, the serum homocysteine level was negatively correlated with adaptive behavior DQ (*r*_s_ = −0.181, *p* = 0.05) and gross motor DQ (*r*_s_ = −0.216, *p* = 0.019). Serum folate levels were positively correlated with adaptive behavior DQ (*r*_s_ = 0.365, *p* < 0.001), gross motor DQ (*r*_s_ = 0.349, p < 0.001), fine motor DQ (*r*_s_ = 0.211, *p* = 0.027), language DQ (*r*_s_ = 0.229, *p* = 0.016), and personal–social DQ (*r*_s_ = 0.186, *p* = 0.052). Serum vitamin B12 levels were positively correlated with adaptive behavior DQ (*r*_s_ = 0.323, *p* = 0.001), gross motor DQ (*r*_s_ = 0.246, *p* = 0.009), and language DQ (*r*_s_ = 0.192, *p* = 0.044). The associations between serum homocysteine, folate, and vitamin B12 levels and adaptive behavior, gross motor, fine motor, language, and social behavior DQs in the multiple linear regression model are shown in Supplementary Table 3 and Figure 7. Serum folate (β = 8.687, 95% CI: 0.217~17.158) and vitamin B12 (β = 8.320, 95% CI: 0.940~15.70) levels were significantly positively related to the adaptive behavior DQ. No significant association was found between homocysteine and Gesell DQ scores. Stratified analyses by age showed that serum vitamin B12 was positively related to adaptive behavior (β = 16.476, 95% CI: 5.076~27.876) and language (β = 15.186, 95%CI: 1.581~28.791) DQs in children aged >3 years (Supplementary Table 4).

## Discussion

Numerous epidemiological studies reported a connection between homocysteine/folate/vitamin B12 and ASD, but there has been a lack of adequate controls for confounding variables, a large sample size, and rigorous statistical analyses that might influence the interpretation of the results. As far as we know, this is the first study to comprehensively evaluate the interrelations between serum homocysteine, folate, and vitamin B12 levels and their associations with the clinical outcomes and severity of ASD after adjusting for known confounding variables such as age, sex, BMI, use of vitamins, and dietary preference. We further confirmed whether this association between homocysteine/folate/vitamin B12 levels and ASD is linear or nonlinear.

Our findings from this study strengthen the evidence that increased homocysteine is associated with ASD linearly and may serve as a novel and sensitive biomarker for distinguishing children with ASD from healthy subjects. The finding that serum homocysteine was higher in children with ASD was robust after adjustment for relevant covariates, which was in line with a recently published meta-analysis that examined 31 articles involving 3304 subjects (Guo et al., 2020a). The difference in serum homocysteine levels between children with ASD and healthy controls was stable and was not changed by clinical characteristics such as age, sex, or eating habits. In addition, our study revealed that homocysteine showed a good diagnostic ability to discriminate children with ASD from healthy controls. Overall, homocysteine could be considered a stable biomarker to predict ASD risk prior to the onset of behavioral abnormalities. To date, few epidemiological studies focused on the specific shape of relationships between homocysteine and ASD. Our results identified a linear association between homocysteine and ASD, which could be in part explained by previously reported *in vitro* experiments showing that higher homocysteine levels exhibited neurotoxicity in a dose-dependent manner (Fan et al., 2017). Regarding the potential biological mechanisms supporting these findings, emerging evidence suggests that impaired homocysteine metabolism is linked to induced oxidative stress, mitochondrial impairment, and methylation impairment, all of which are involved in autism pathology (Rossignol and Frye, 2014). Moreover, elevated homocysteine levels have been found to show toxic effects on the nervous system and accumulate in the brain of animals, resulting in abnormal brain energy metabolism, neural or cognitive dysfunction, and behavioral alterations (Dos Santos et al., 2019; Wyse et al., 2020).

On the other hand, the optimal cutoff value for serum homocysteine levels to distinguish ASD was 6.69 μmol/L based on the Youden index. As is well known, the cutoff value of 15 μmol/L for serum homocysteine levels is used to define hyperhomocysteinemia (>15 μmol/L) in adults (Yang et al., 2014), but this is not suitable for children because serum homocysteine levels may differ between age groups (Setién-Suero et al., 2016). We also observed an age difference in serum homocysteine levels of the children with ASD. There is currently no accepted cutoff value for elevated homocysteine levels in children, but our results provide some evidence for developing age-dependent homocysteine cutoff values.

Because serum folate and vitamin B12 levels are the important determinants of homocysteine levels in children (González-Gross et al., 2012) and are negatively associated with homocysteine levels, which was also verified in our studies, we examined the association of folate and vitamin B12 levels with ASD. However, the finding that serum folate and vitamin B12 levels were lower in the children with ASD did not hold after additional adjustment for the combined effects of homocysteine/folate/vitamin B12 levels, which was in contrast to previous research (Zhu et al., 2020). It is worth noting that higher homocysteine levels were strongly and independently associated with ASD, but not with folate and vitamin B12 levels, which could be explained by several mechanisms involved in the disruption of homocysteine metabolism. One intriguing possibility is that cerebral folate deficiency under normal blood folate levels caused by the impaired transport of folate across the blood–brain barrier (Frye et al., 2013c; Ramaekers et al., 2020), leads to homocysteine accumulation (Ramaekers et al., 2014). Another possible reason is that homocysteine levels are strictly controlled by two metabolic pathways (Azzini et al., 2020), of which dysfunction in folate-independent remethylation and transsulfuration pathways, but not in folate and vitamin B12-dependent pathways, leads to elevated serum homocysteine concentrations. Human and animal experiments have reported an association between dysfunction in folate-independent pathways and increased homocysteine levels. One experiment in an animal model of ASD showed elevated serum homocysteine concentrations due to decreased betaine homocysteine methyltransferase expression, which is one of the folate-independent remethylation-related enzymes (Huang et al., 2019). An open-label trial in children with ASD who were treated with folic acid and methylcobalamin showed that excess intake of folate and vitamin B12 did not significantly change homocysteine levels (James et al., 2009). Other factors like MTHFR gene mutations can also induce elevated homocysteine without folate or B12 deficiency in children with ASD (Paşca et al., 2009).

Only a few studies examined the association between serum homocysteine, folate, and vitamin B12 and the severity of ASD, as evaluated by CARS scores, and these studies consistently lacked clinical characteristics and nutritional factors as control covariates. Some studies (Han et al., 2015; Altun et al., 2018), but not all (Guo et al., 2020b; Li et al., 2021), showed that high levels of homocysteine and low levels of folate and vitamin B12 are associated with ASD severity. Such data would be of interest to determine if the three metabolites can be used as markers for ASD severity. Our analyses demonstrated that children with severe ASD symptoms had a higher mean homocysteine level but a lower mean folate level than children with mild ASD symptoms, but no significant difference in serum vitamin B12 levels was observed between the two groups. Moreover, we also observed a negative association between folate levels and the severity of ASD independent of confounding factors. Recent findings of greater improvement in autism symptoms following folate supplementation in children with ASD partly support our results (Sun et al., 2016; Hoxha et al., 2021). In addition, ASD symptoms may vary between age groups (Guthrie et al., 2013), and serum folate levels were associated with age and were relatively higher in children aged ≤ 3 years, which was consistent with some previous studies (González-Gross et al., 2012; Li et al., 2021). Our age-stratified analysis suggested that folate deficiency at younger ages might lead to a higher risk of severe ASD, which might be associated with the role of folate in the formation of synapses, which peaks before the age of 3 years (Nelson, 2007). Furthermore, we observed a strong, linear, and independent association between low folate levels and ASD severity in RCS models, indicating that serum folate might act as a predictor for ASD severity. An *in vitro* study has also suggested that folate had a dose-dependent protective effect on neurons (Chen et al., 2013). In summary, although no significant differences in folate levels between children with ASD and healthy children were found in our study, children with ASD had folate deficiencies on a larger scale, which were related to autism symptoms. Thus, high folate concentrations, especially in children younger than 3 years, may be a protective factor against autism.

Previous epidemiological research mainly explored the relationship between homocysteine, folate, and vitamin B12 and neurodevelopment in children with ASD using correlation analyses (Guo et al., 2020b; Ljungblad et al., 2021). For example, a previous study found that high homocysteine levels were associated with motor scores using the Ages and Stages Questionnaire in presumed healthy infants aged 3–7 months (Ljungblad et al., 2021), which was in line with our results. Another study found that folate levels were positively correlated with Gesell DQ scores in children with ASD, but vitamin B12 levels were not significantly correlated with neurodevelopment in children with ASD (Guo et al., 2020b). However, some studies reported that decreased serum vitamin B12 levels were correlated with neurodevelopment disorders (Kvestad et al., 2017; Hope et al., 2020). These inconsistent results seem to be related to heterogeneity in the methodological design. Thus, by using a multiple linear regression model, we further demonstrated that both serum folate and vitamin B12 levels were positively associated with adaptive behavior in children with ASD. Well-designed clinical trials have also indicated that supplementation with vitamin B12 and folate can be beneficial for neurodevelopment in children with ASD, especially in adaptive behavior (Frye et al., 2013b; Rossignol and Frye, 2021b). Folate and vitamin B12 are crucial for normal neuron function, and serious deficiencies of these vitamins might directly affect brain function by disrupting myelination and synapse formation during neural development in the brain (Prado and Dewey, 2014). Notably, a higher vitamin B12 level at age >3 years was associated with higher adaptive behavior DQ and language DQ in children with ASD. James et al. also reported that vitamin B12 supplementation in children with ASD aged >3 years is effective in improving autism symptoms (Adams et al., 2018). However, larger studies in future are needed to verify these associations. This finding suggests that serum folate and vitamin B12 concentrations are closely related to cognitive functioning.

The major strength of our study was that multiple variable regression models with confounder control were used to assess the associations between serum homocysteine, folate, and vitamin B12 levels and the outcomes and severity of ASD. Moreover, RCS regression was used to analyze the dose–response relationships of serum homocysteine, folate, and vitamin B12 with ASD. In addition, diverse statistical methods were applied to examine the utility of the three parameters as multidimensional biomarkers for predicting ASD risk prior to the onset of behavioral abnormalities or for predicting the developmental trajectory of children with ASD. However, there are some limitations to this study. First, this study was a case–control study and could not directly analyze the relative risk, thus limiting the attribution of causality in the results. Second, the sample numbers were limited in the stratified analysis (sex, BMI, and dietary habits); thus, the statistical efficiency was reduced and multivariate analyses could not be performed. Thus, further longitudinal studies with larger sample sizes and functional experiments are necessary to verify the results of this study. Third, there are still some confounding factors that were not considered, such as genetic factors, types of vitamin supplementation, and drug treatments, which might have biased our results. Therefore, we cannot rule out residual confounding by unmeasured factors.

In summary, this study showed that an increased homocysteine level was associated with the risk of ASD in a linear manner and is thus a novel diagnostic biomarker for ASD. Decreased concentrations of folate and vitamin B12 were associated with poor clinical profiles of children with ASD. Our findings suggest that serum homocysteine, folate, and vitamin B12 can be used as biomarkers to predict the development profile and clinical severity of children with ASD. Homocysteine-lowering interventions or folate and vitamin B12 supplementation might be a viable treatment strategy for ASD. However, additional research needs to confirm the observed associations and to clarify if they may serve as new therapeutic targets for ASD.

## Data availability statement

The original contributions presented in the study are included in the article/Supplementary material, further inquiries can be directed to the corresponding author.

## Ethics statement

The studies involving human participants were reviewed and approved by the Ethics Committee of the Third Affiliated Hospital of Zhengzhou University. Written informed consent to participate in this study was provided by the participants' legal guardian/next of kin. Written informed consent was obtained from the minor(s)' legal guardian/next of kin for the publication of any potentially identifiable images or data included in this article.

## Author contributions

CZ conceptualized and designed the study and critically reviewed and revised the manuscript. BL conceptualized and designed the study, performed the statistical analysis, and drafted the initial manuscript. YX and QZ conducted data analyses and blood measurements. DP, LZ, ML, WL, and GD were involved in the collection of blood and clinical data. All authors read and approved the final manuscript.

## Funding

This work was supported by the National Nature Science Foundation of China (81902162, U21A20347), the Swedish Research Council (2018-02267), Swedish governmental grants to scientists working in healthcare (ALFGBG-965197), the Henan Provincial Science and Technology Department (222102310161, 182102310688, 202102310221), and the Henan Province Medical Research Project (LHGJ20190349).

## Conflict of interest

The authors declare that the research was conducted in the absence of any commercial or financial relationships that could be construed as a potential conflict of interest.

## Publisher's note

All claims expressed in this article are solely those of the authors and do not necessarily represent those of their affiliated organizations, or those of the publisher, the editors and the reviewers. Any product that may be evaluated in this article, or claim that may be made by its manufacturer, is not guaranteed or endorsed by the publisher.
