# Supplementary material for: Interrelation between homocysteine metabolism and the development of autism spectrum disorder in children
Source: Front Mol Neurosci. 2022 Aug 15;15:947513. doi: 10.3389/fnmol.2022.947513 (PMC9421079; doi:10.3389/fnmol.2022.947513)
Supplement: Supplementary file 1 [file Data_Sheet_1.docx]

Supplementary Figures


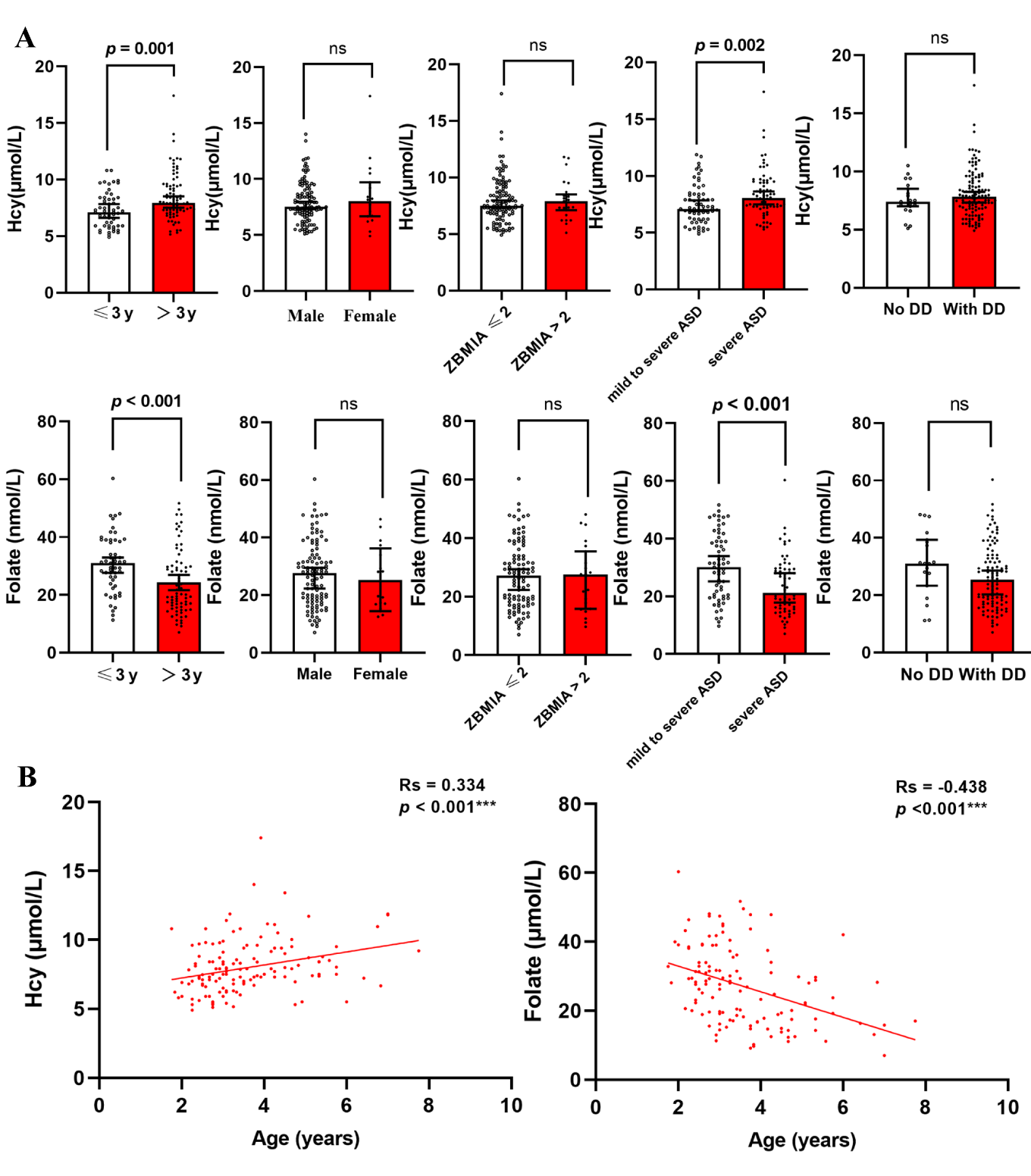


**Supplementary Figure 1.** Impact of gender, age, BMI, ASD severity and comorbidity on serum homocysteine (Hcy) and folate levels in children with ASD (A) and correlations between serum Hcy, folate and age (B). Results are presented as Spearman correlation coefficients (*r*_s_) and p-values. DD, developmental delay. “ns” means no significant difference.


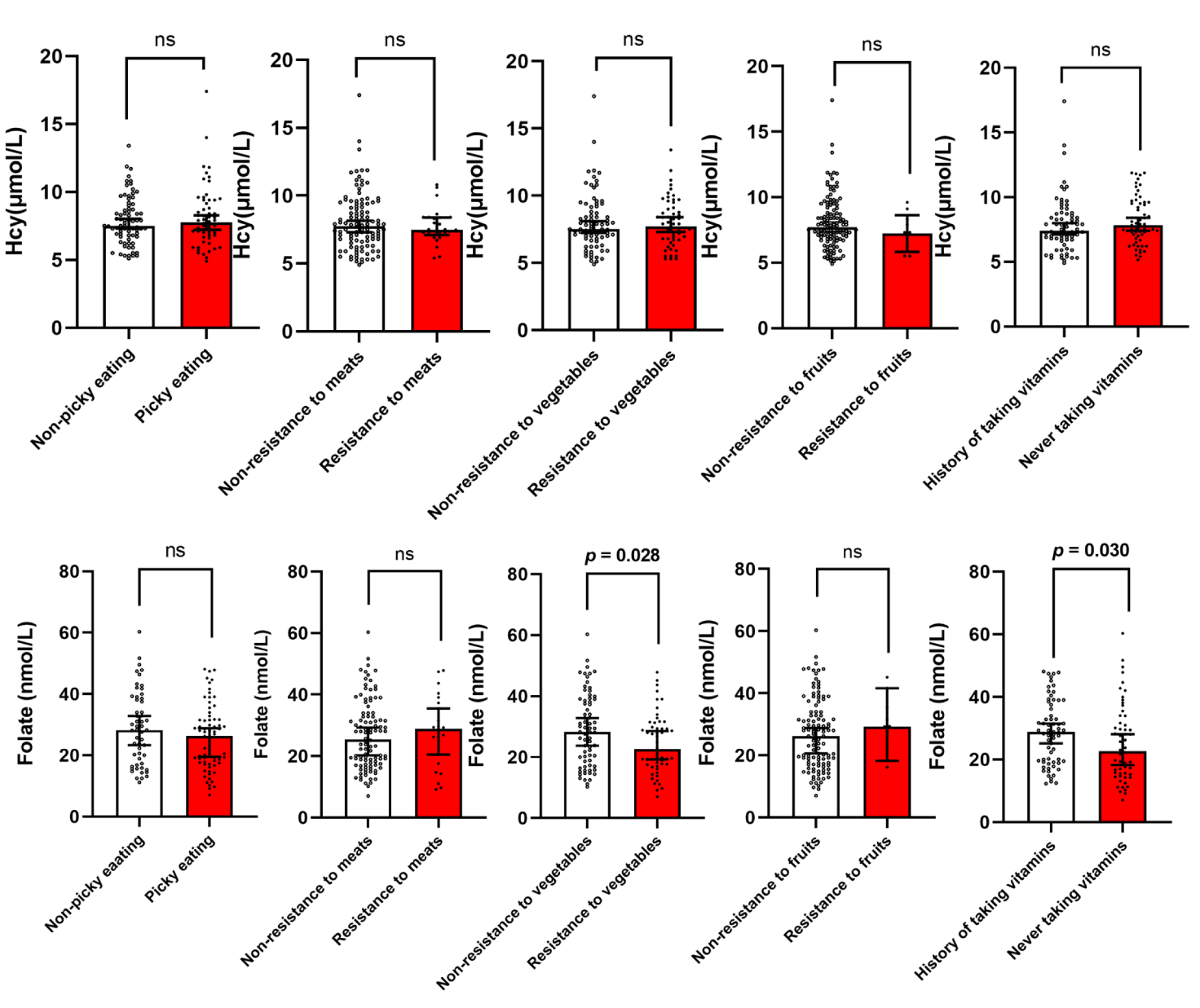
**Supplementary Figure 2.** Impact of dietary preference for meat and vegetables and use of vitamins on serum homocysteine (Hcy) and folate levels in children with ASD. “ns” means no significant difference.


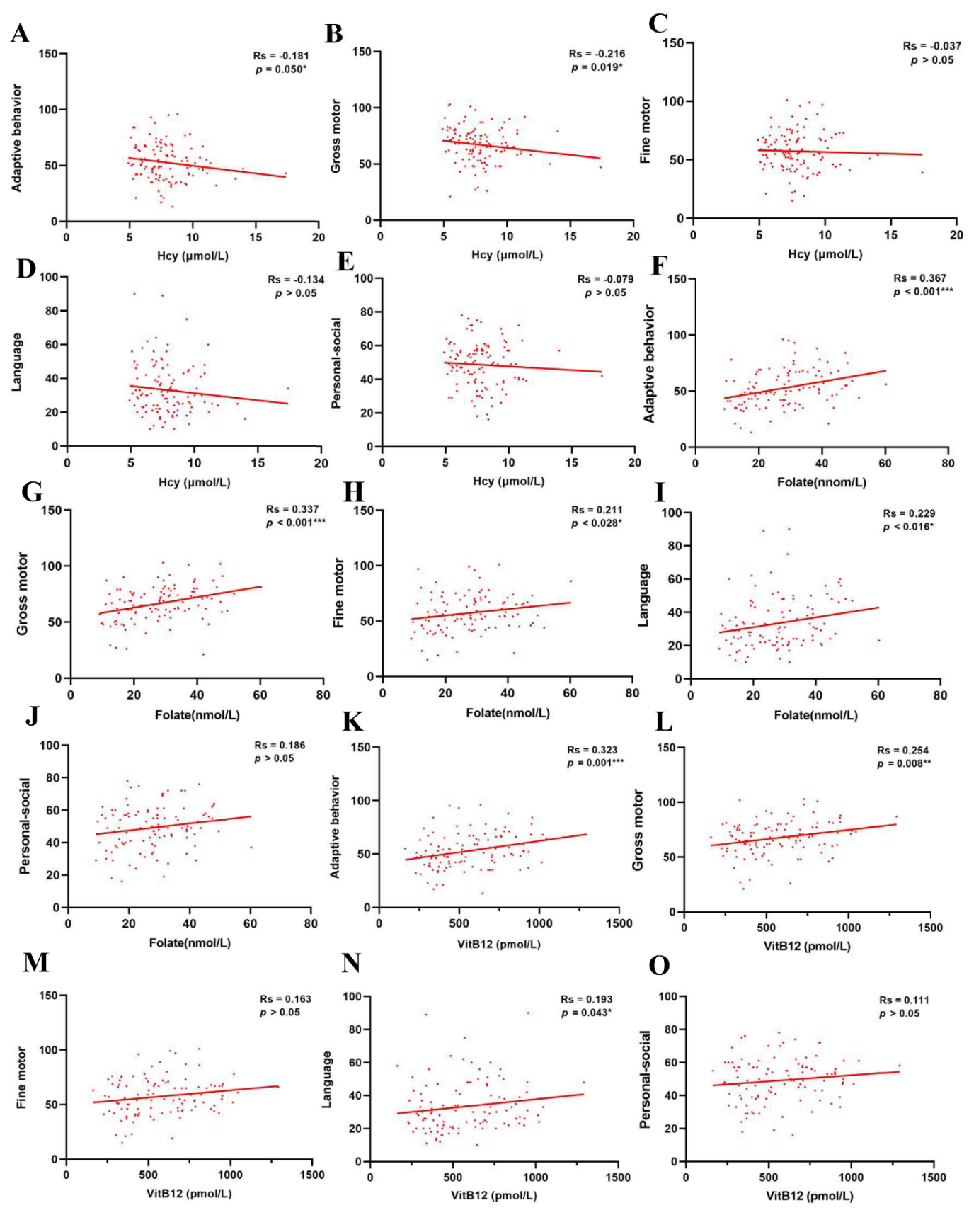
**Supplementary Figure 3.** Effect of serum homocysteine (Hcy) (A–E), folate (F–J), and vitamin B12 (VitB12) (K–O) levels on the GDS scores of children with ASD. Results are presented as Spearman correlation coefficients (*r*_s_) and p-values.

## Supplementary tables

**Supplementary Table 1.** Multivariate linear logistic regression model for comparison of ln-transformed serum homocysteine, folate, and vitamin B12 levels between children with ASD and healthy controls

|  | **Crude ^a^** | | **Model 1 ^b^** | **Model 2 ^c^** |
| --- | --- | --- | --- | --- |
|  | **Controls**  **(n = 84)** | **ASD**  **(n = 135)** | **ASD**  **(n = 135)** | **ASD**  **(n = 135)** |
|  | **β (95%CI)** | **β (95%CI)** | **β (95%CI)** | **β (95%CI)** |
| Hcy | 1.00 (ref) | 0.392 (0.327, 0.457)  *p* < 0.001 | 0.370 (0.299, 0.441)  *p* < 0.001 | 0.299 (0.235, 0.363)  *p* < 0.001 |
| Folate | 1.00 (ref) | –0.254 (–0.379, –0.130)  *p* < 0.001 | –0.238(–0.369, –0.107)  *p* < 0.001 | 0.078(–0.060, 0.217)  *p* = 0.267 |
| VitB12 | 1.00 (ref) | –0.201(–0.325, –0.078)  *p* = 0.002 | –0.188(–0.327, –0.048)  *p* = 0.009 | 0.025(–0.134, 0.184)  *p* = 0.756 |

Data are presented as β (95%CI). ASD, autism spectrum disorder. Hcy, homocysteine. VitB12, vitamin B12. β (95%CI), regression coefficient (95% confidence interval). Univariate linear regression was used for the unadjusted (crude) model, and multivariate linear regression was used for the adjusted model.

a, no adjustment.

b, adjusted for age, sex, BMI, picky eating, and use of vitamins

c, adjusted for age, sex, BMI, picky eating, use of vitamins, and the other two metabolites.

**Supplementary Table 2.** Adjusted multiple logistic regression for the severity of ASD associated with quartiles of ln-transformed folate levels stratified by age

| **Subgroup** | **Folate, Quartiles** | | | |
| --- | --- | --- | --- | --- |
| **Age (years)** | **Q1 (≤ 19.545)** | **Q2 (19.545**–**29.270)** | **Q3 (29.270**–**37.70)** | **Q4 (> 37.70)** |
|  | **OR (95%CI)** | **OR (95%CI)** | **OR (95%CI)** | **OR (95%CI)** |
| Overall  (n = 135) | 4.227 (1.022, 17.49) | 2.798 (0.769, 10.18) | 1.807 (0.499, 6.543) | 1.00 (Ref) |
| p-value | 0.038 | 0.104 | 0.343 | - |
| ≤ 3  (n = 55) | 14.381 (0.956, 216.25) | 4.982 (0.817, 30.39) | 1.973 (0.293, 13.29) | 1.00 (Ref) |
| p-value | 0.054 | 0.082 | 0.485 | - |
| > 3  (n = 80) | 9.765 (0.938, 101.61) | 5.604 (0.483, 64.98) | 7.715 (0.586, 87.85) | 1.00 (Ref) |
| p-value | 0.057 | 0.168 | 0.123 | - |

Data are presented as adjusted OR (95%CI). OR (95%CI), Odds ratio (95% confidence interval). Adjusted factors were age, sex, BMI, picky eating, use of vitamins, and serum homocysteine and vitamin B12 levels.

**Supplementary Table** **3.** Adjusted multiple linear regression for adaptive behavior, gross motor, fine motor, language, and personal-social behavior development quotient with ln-transformed serum homocysteine, folate, and vitamin B12 concentrations

| **GDS** | **Hcy (n = 135)** | **Folate (n = 135)** | **VitB12 (n = 135)** |
| --- | --- | --- | --- |
|  | **β (95%CI)** | **β (95%CI)** | **β (95%CI)** |
| Adaptive behavior | 8.466 (-6.70, 23.633)  *p* = 0.271 | 8.687 (0.217, 17.158)  *p* = 0.045 | 8.320 (0.940, 15.70)  *p* = 0.028 |
| Gross motor | 0.986 (-12.404, 14.375)  *p* = 0.884 | 7.187 (-0.291, 14.665)  *p* = 0.059 | 1.820 (-4.695, 8.336)  *p* = 0.580 |
| Fine motor | 14.987 (-1.394, 31.367)  *p* = 0.072 | 6.546 (-2.518, 15.609)  *p* = 0.155 | 4.754 (-3.374, 12.882)  *p* = 0.248 |
| Language | -3.652 (-19.732, 12.429)  *p* = 0.653 | 6.250 (-2.732, 15.231)  *p* = 0.170 | 3.886 (-3.939, 11.711)  *p* = 0.327 |
| Personal-social | 8.242 (-4.745, 21.230)  *p* = 1.260 | 4.738 (-2.855, 11.611)  *p* = 0.232 | 1.575 (-4.791, 7.941)  *p* = 0.624 |

Data are presented as β (95%CI). GDS, Gesell Developmental Schedules; β (95%CI), regression coefficient (95% confidence interval); Hcy, homocysteine. VitB12, vitamin B12. Adjusted for age, sex, BMI, picky eating, use of vitamins, and the other two metabolites.

**Supplementary Table 4.** Adjusted multiple linear regression for gross motor, fine motor, adaptive behavior, language, and social behavior development quotients with ln-transformed folate and vitamin B12 concentrations stratified by age

| **GDS** | **≤ 3years (n = 55)** | | **> 3 years (n = 80)** | |
| --- | --- | --- | --- | --- |
|  | **β (95%CI)** | **p-value** | **β (95%CI)** | **p-value** |
| **Adaptive behavior** |  | | | |
| Folate | –1.040 (–17.06, 14.980) | 0.896 | 6.931 (–2.428, 16.290) | 0.143 |
| VitB12 | 11.297 (–0.957, 23.55) | 0.070 | 16.476 (5.076, 27.876) | 0.006 |
| **Gross motor** |  | | | |
| Folate | –4.811 (–16.59, 6.963) | 0.413 | 7.893 (–2.754, 18.539) | 0.142 |
| VitB12 | 7.257 (–1.749, 16.263) | 0.111 | 6.054 (–6.914, 19.022) | 0.352 |
| **Fine motor** |  | | | |
| Folate | 1.280 (–13.748, 16.309) | 0.864 | 7.509 (–4.831, 19.848) | 0.227 |
| VitB12 | 4.389 (–6.889, 15.666) | 0.435 | 10.342 (–4.688, 25.373) | 0.173 |
| **Language** |  | | | |
| Folate | –13.24 (–27.367, 0.885) | 0.065 | 3.585 (–7.584, 14.755) | 0.521 |
| VitB12 | –2.95 (–13.755, 7.855) | 0.584 | 15.186 (1.581, 28.791) | 0.029 |
| **Personal-social** |  | | | |
| Folate | –8.223 (–20.825, 4.379) | 0.194 | 7.007 (–2.458, 16.471) | 0.143 |
| VitB12 | 4.057 (–5.571, 13.685) | 0.399 | 9.204 (–2.325, 20.732) | 0.115 |

GDS, Gesell Developmental Schedules. VitB12, vitamin B12. β (95%CI), regression coefficient (95% confidence interval). Adjusted for age, sex, BMI, picky eating, use of vitamins, and the other two metabolites.
